# Supplementary material for: The Physiological Response of Different Brook Willow (Salix acmophylla Boiss.) Ecotypes to Salinity
Source: Plants (Basel). 2022 Mar 10;11(6):739. doi: 10.3390/plants11060739 (PMC8953935; doi:10.3390/plants11060739)
Supplement: Supplementary file 1 [file plants-11-00739-s001.zip › plants-1614106-supplementary.pdf]

# Supplementary materials

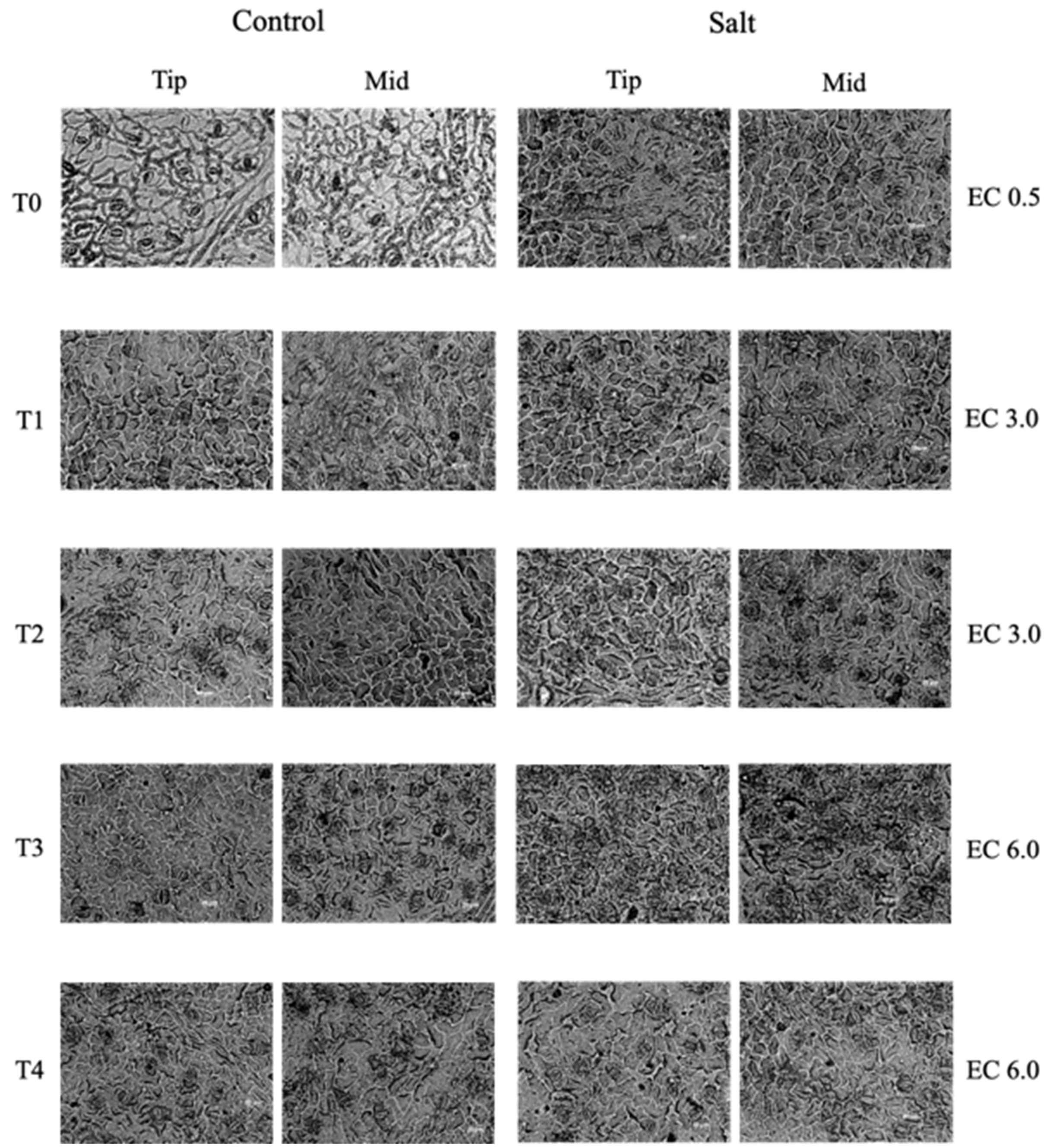


**Figure S1.** Representative photos of epidermal peels of Adom leaves at individual timepoints of from both treatment groups (Control and Salt). Peels were made of the abaxial leaf surfaces at midleaf and leaf tip positions as described in the materials and methods sections. Photographs were taken at 200X with a Zeiss AxioCam camera, model MRm 1.4MP CCD and the AxioVision Imaging System software (Carl Zeiss Microimaging GmbH, Jena, Germany, version 4.9).

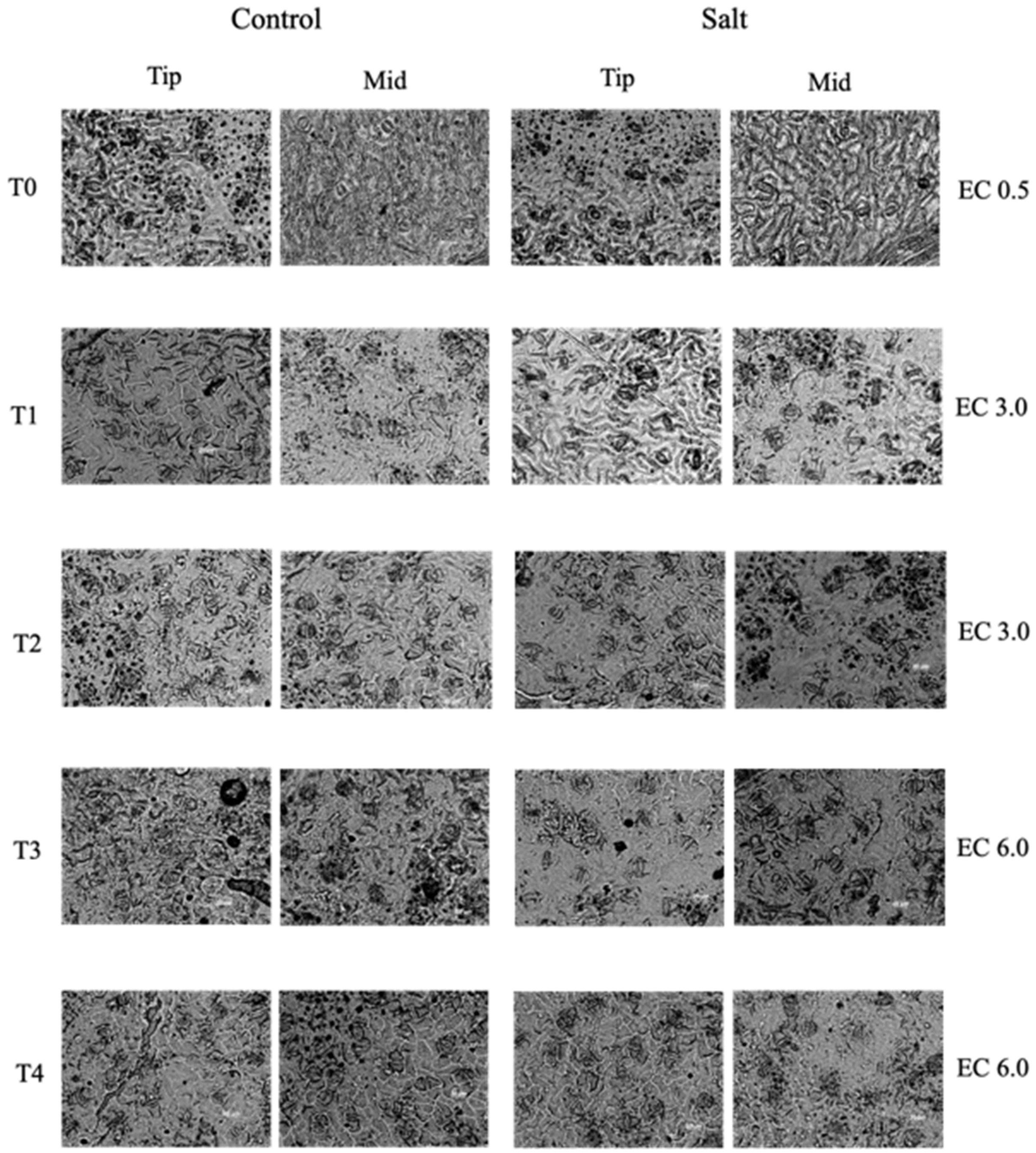


**Figure S2.** Representative photos of epidermal peels of Darom leaves at individual timepoints of from both treatment groups (Control and Salt). Peels were made of the abaxial leaf surfaces at midleaf and leaf tip positions as described in the materials and methods sections. Photographs were taken at 200X with a Zeiss AxioCam camera, model MRm 1.4MP CCD and the AxioVision Imaging System software (Carl Zeiss Microimaging GmbH, Jena, Germany, version 4.9).

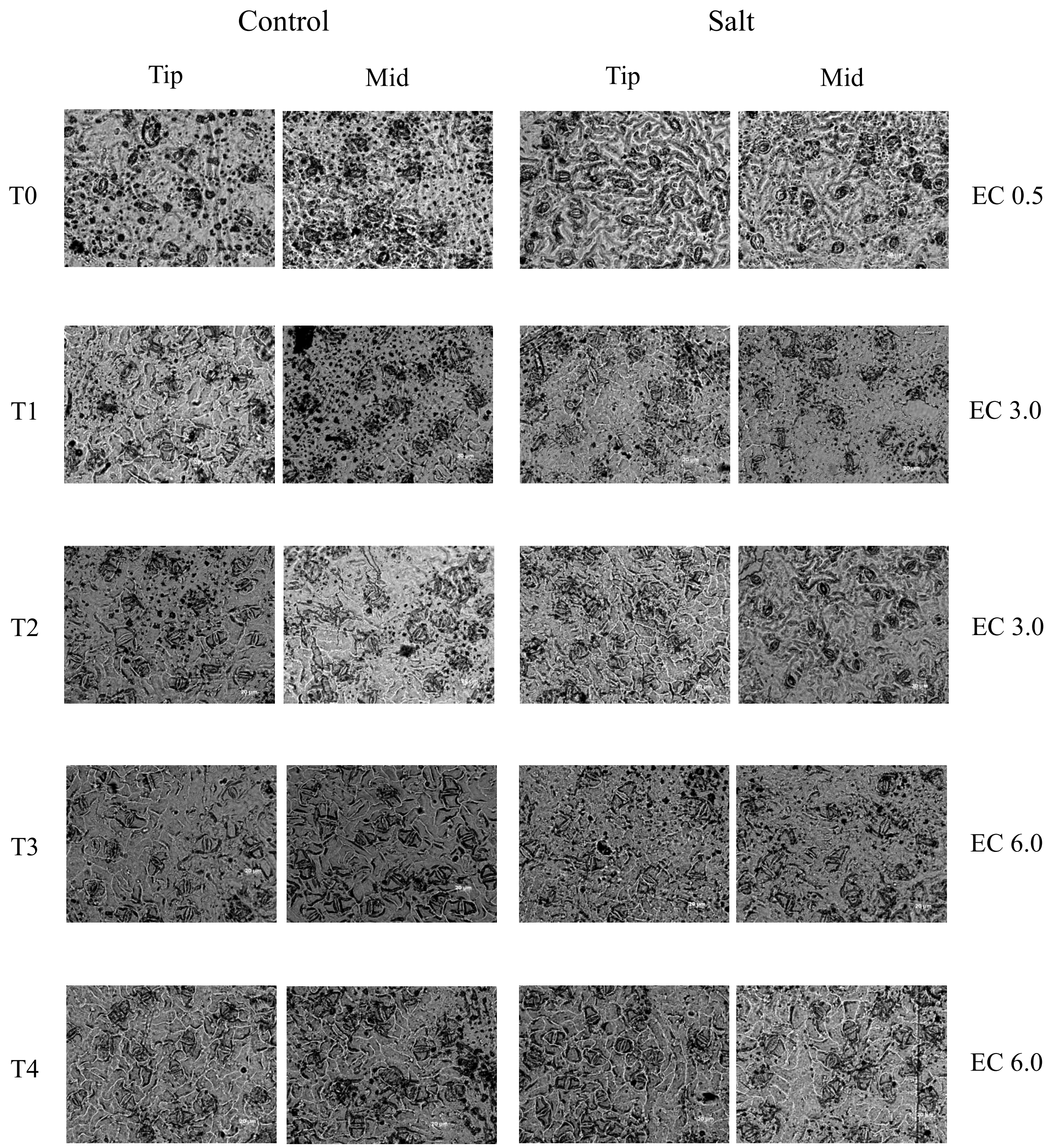


**Figure S3**. Representative photos of epidermal peels of Mea She'arim leaves at individual timepoints of from both treatment groups (Control and Salt). Peels were made of the abaxial leaf surfaces at midleaf and leaf tip positions as described in the materials and methods sections. Photographs were taken at 200X with a Zeiss AxioCam camera, model MRm 1.4MP CCD and the AxioVision Imaging System software (Carl Zeiss Microimaging GmbH, Jena, Germany, version 4.9).

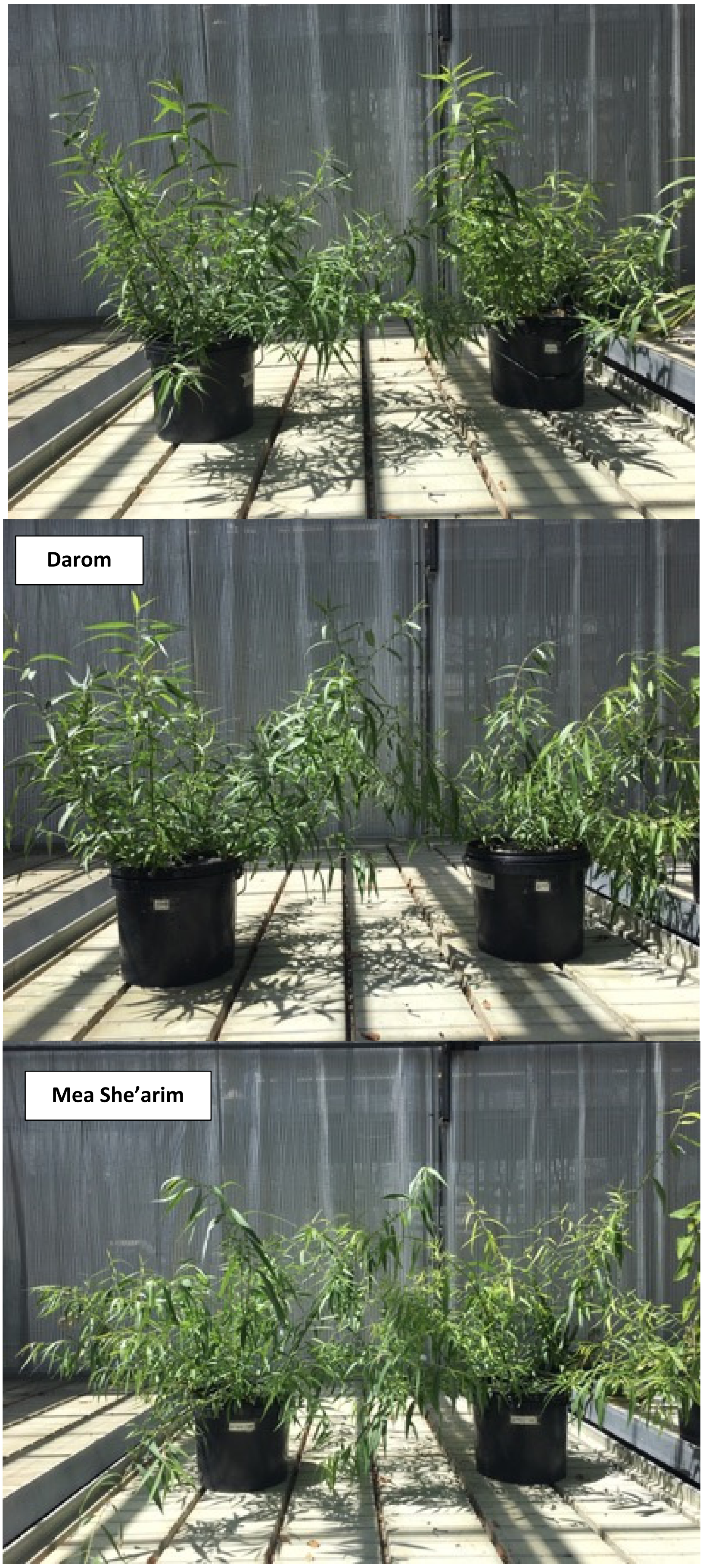


**Figure S4**. The *S.acmophylla* ecotypes used in the current trial under control (left) and salinity (right) conditions before harvesting.

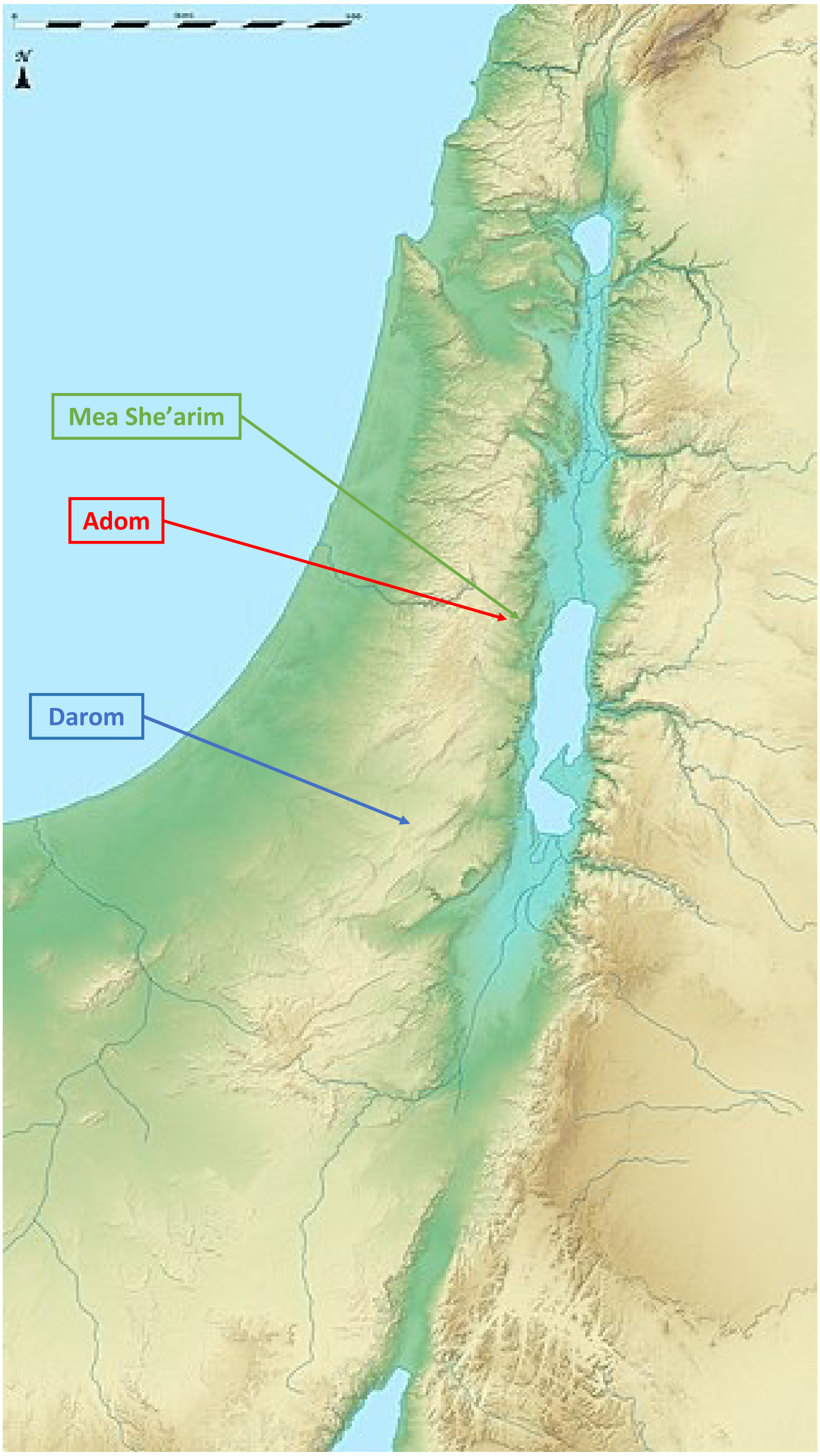


**Figure S5**. Geographic location of different *S.acmophylla* ecotypes used in the current trial.
